# Inflammation and Cardiovascular Cross Talk in Ischemic Vascular Diseases

**DOI:** 10.1155/2017/3161968

**Published:** 2017-03-26

**Authors:** Giorgio Zauli, Veronica Tisato, Joseph D. Raffetto, Mauro Vaccarezza

**Affiliations:** ^1^Department of Morphology, Surgery and Experimental Medicine, University of Ferrara, Ferrara, Italy; ^2^VA Boston HCS, Department of Surgery, Section of Vascular Surgery, Harvard Medical School, Boston, USA; ^3^School of Biomedical Sciences, Faculty of Health Sciences, Curtin University, Perth, WA, Australia

Ischemic vascular diseases include different pathological events characterized by distinctive features but share the common hallmark of inflammation. In this light, myocardial infarction can be a good paradigm to summarize the different connections linking inflammation and the cardiovascular system during an ischemic event. The immune system and inflammation, through several cellular and soluble inflammatory mediators, play a crucial role in the local tissue structural changes of ischemic heart disease, with a different impact and outcome during acute myocardial infarction compared to the more chronic long-term inflammation [1]. In response to acute damage and hemodynamic stress, there is expansion of resident immune cells and recruitment of extra cells involved in a critical cross talk with parenchymal cells [2, 3]. In other words, postischemic tissue repair is crucial to survival. Recruited inflammatory cells can remove debris and facilitate the repair process; conversely, unrestrained inflammation inhibits optimal healing leading to adverse events. Moreover, other mediators such as some key coagulation factors might influence innate immunity as well as cell-mediated reactions like healing, response to tissue injury, or inflammatory processes [4, 5]. Overall, as recently suggested, the different immune/inflammatory cell subsets act as messengers implicated in novel inflammatory networks that link different organ systems enlarging the continuum beyond the myocardium and blood vessels in a more integrative pathophysiology standpoint [6].

This special issue aims to collect insights about this cross talk with a dual purpose: on the one hand to expand the comprehension on the mechanisms of action and impact of “old” inflammatory mediators and on the other to bring out “new” potential pathways and intermediates. The overall aim is to increase knowledge on the pathophysiological processes of ischemic vascular disease to improve diagnosis and treatment.

The first set of articles draws attention to soluble and cellular circulating mediators with a role in vascular inflammation. M. L. Morieri et al. have considered an “old” inflammatory factor such as interleukin 6 (IL-6) that shows different effects on the acute inflammatory responses compared with that of chronic low-grade systemic inflammation. In particular, since the “transsignalling” mediated by IL-6 is more linked to the harmful actions of this cytokine especially in the cardiovascular setting, the specific targeting of this pathway by using inhibitors such as soluble glycoprotein 130 might be a promising therapeutic strategy. The article of B. Toffoli et al. addresses the role of two other cytokines belonging to the TNF family. Osteoprotegerin (OPG) and TNF-related apoptosis-inducing ligand (TRAIL) are two factors showing controversial effects on several physiopathological contexts [7, 8]. In a preclinical model, the authors show that dyslipidaemia and diabetes, two risk factors for cardiovascular disease, modify the vascular and cardiac expression of OPG and TRAIL leading to an increased OPG/TRAIL ratio. This alteration could contribute to the changes in circulating OPG/TRAIL ratio observed in patients with diabetes and cardiovascular disease, and they could mediate/contribute to atherosclerosis development and cardiac remodelling. As far as cellular mediators, H. Li et al. have addressed the role of neutrophil granulocytes in the pathophysiology of vascular disease in a wide cohort of patients undergoing chronic haemodialysis. In the same line, R. Helseth et al. deal with neutrophil cell activation in acute myocardial infarction. The profile and role of neutrophil extracellular traps (NET), namely, spindle-like networks made in the extracellular space by neutrophil, have been addressed in patients with ST-elevation myocardial infarction suggesting a role of NET in acute and chronic atherothrombosis in line with previous studies [9, 10]. Finally, W. Zhong et al. have a methodological and “laboratory-oriented” approach and propose an experimental method to evaluate the role of CD137 (41-BB) signalling in the pathogenesis of the atherosclerotic plaque and vascular damage.

Other authors have indeed contributed to the main topic of the special issue by exploring possible mechanisms underlying the pathophysiology of ischemic diseases. S. Wang et al. and M. Neri et al. address ischemia reperfusion injury, one of the Achilles heels of the current therapy for ischemic vascular diseases. In their work, S. Wang et al. highlight autophagy as a fundamental mechanism involved in ischemic pathology in an experimental model of diabetes. In the same line but from a different perspective, M. Neri et al. review the current literature on the ischemia reperfusion injury following myocardial infarction, highlighting the relevance of a calpain system, oxidative and NO-linked pathways, and ventricular remodelling discussing the evidences from a medico-legal standpoint to better understand sudden deaths following myocardial infarction [11, 12]. Finally, M. V. Arcidiacono et al. change perspective and address the cross talk between cardiovascular and kidney systems showing that patients with chronic kidney disease are prone to develop cardiovascular events in a mutual relationship. Indeed, they demonstrate that the higher incidence of microangiopathy of the common carotid artery wall drives the higher incidence of atheromatous disease in these patients.

In conclusion, we are pleased to introduce the reader to this translational research topic on inflammation and vascular cross talk in ischemic diseases. We hope that the research articles/reviews selected and presented might be an opportunity for further discussions and future deeper investigations to move forward by improving knowledge in the field, with the overall aim of increasing the available diagnostic and therapeutic options in the context of ischemic diseases.
